# Factors influencing adherence to non-communicable disease medication in India: secondary analysis of cross-sectional data from WHO - SAGE2

**DOI:** 10.3389/fphar.2023.1183818

**Published:** 2023-10-13

**Authors:** Abraham Tolley, Kirpal Grewal, Alessa Weiler, Anna Maria Papameletiou, Refaat Hassan, Saurav Basu

**Affiliations:** ^1^ School of Clinical Medicine, University of Cambridge, Cambridge, England; ^2^ Faculty of Natural Science, University of Cambridge, Cambridge, England; ^3^ Indian Institute of Public Health, Public Health Foundation of India, New Delhi, India

**Keywords:** medication adherence, medication compliance, India, WHO, non-communicable disease (NCD), chronic diseases, multimorbidity, SAGE2

## Abstract

**Background:** Non-communicable diseases (NCDs) are a leading cause of death globally and disproportionately affect those in low- and middle-income countries lower-middle-income countries. Poor medication adherence among patients with NCDs is prevalent in India due to lack of initiation, missed dosing or cessation of treatment, and represents a growing healthcare and financial burden.

**Objective:** This study aimed to identify factors influencing medication adherence in adults with NCDs in India.

**Methods:** We performed a cross-sectional study, conducting secondary data analysis on the second wave of the World Health Organisation’s ‘Study on global AGEing and adult health (SAGE)’, a survey that collected data from predominantly older adults across India. Bivariate analysis and multivariate logistic regression modelling were conducted to specifically interrogate the reasons for lack of initiation and cessation of treatment. Reporting of this study was informed by the STROBE guidelines.

**Results:** The average medication adherence rate was 51% across 2,840 patients with one or more NCDs, reflecting non-initiation and lack of persistence of treatment. The strongest factor significantly predicting non-adherence to medication across these components was multimorbidity (odds ratio 0.47, 95% CI 0.40–0.56). Tobacco use (OR = 0.76, CI 0.59–0.98) and never having attended school (OR = 0.75, CI 0.62–0.92) were significantly associated with poor medication adherence (*p* < 0.05) while rural living (OR = 0.70, CI 0.48–1.02), feelings of anxiety (OR = 0.84, CI 0.66–1.08) and feelings of depression (OR = 0.90, CI 0.70–1.16) were factors lacking statistically significant association with medication adherence on multivariate analysis. Older age (OR = 2.02, CI 1.51–2.71) was significantly associated with improved medication adherence whilst there was a weak association between increased wealth and improved medication use.

**Limitations:** The SAGE2 survey did not capture whether patients were taking their medication doses according to prescribed instructions—as a result our findings may under-estimate the true prevalence of medication non-adherence.

**Conclusion:** Our analysis provides evidence that poor medication adherence in India is multifactorial, with distinct socioeconomic and health-system factors interacting to influence patient decision making. Future large-scale surveys interrogating adherence should assess all components of adherence specifically, whilst public health interventions to improve medication adherence should focus on barriers that may exist due to multimorbidity, comorbid depression and anxiety, and low educational status.

## Introduction

Non-communicable diseases (NCDs) are chronic health conditions which represent leading causes of death globally, disproportionately affecting those in low- and middle-income countries (LMICs) where more than 75% of NCD deaths occur (World Health Organisation, 2022a). In India, there is a growing burden of NCDs where (as of 2016) cardiovascular disease, respiratory disease and diabetes kill around 4 million people annually and, for the most part, prematurely (Arokiasamy, 2018).

Treatment of NCDs commonly involves long-term medication use - however approximately 50% of patients do not take their medications as prescribed (World Hea lth Organisation, 2003). Medication adherence is understood to include the initiation (taking the first dose of prescribed medication), implementation (the extent to which a patient’s dosing corresponds to the prescribed instructions) and discontinuation (patient stopping taking the treatment) components according to standard taxonomy, while persistence is defined as the time between initiation until the last dose is taken (Vrijens et al., 2012).

Barriers to medication adherence involve both patient- and physician-related factors that influence behaviour as well as system-related factors that limit access to pharmacotherapy (Brown and Bussell, 2011). These system-related barriers to medication access are complex and often relate to public health factors including provider availability, cultural and language barriers, and health literacy (Pharmacy Quality Alliance, 2019). Poor adherence to medication treatment, is associated with adverse patient outcomes, increased patient mortality, and consequently increased patient and healthcare system costs (Cutler et al., 2018; Walsh et al., 2019). The escalating burden of NCDs in lower-middle-income countries (LMICs) driven by an ongoing epidemiological and demographic transition, with concomitant low medication adherence, increases the propensity of premature onset, progression, and risk of death (Boutayeb and Boutayeb, 2005; Asogwa et al., 2022). Consequently, improving medication adherence is considered one of the biggest public health challenges by the World Health Organization (WHO) (World Hea lth Organisation, 2003; World Health Organization WHO, 2020).

Non-adherence is prevalent across chronic NCDs in India, with low adherence and multiple barriers to adherence reported in multiple studies examining NCDs including cardiovascular diseases, diabetes, and depression (Dalal et al., 2021; Pillai et al., 2021; Krishnamoorthy et al., 2022). Current evidence from India and other LMICs also shows heterogeneity in adherence rates across common NCDs: between 19% and 96% patients are adherent to anti-hypertensive medication based on a systematic review (Dalal et al., 2021), an average of 43.4% (95% confidence interval 17.5%–69.4%) patients adhere to anti-diabetic medications based on a meta-analysis (Azharuddin et al., 2021) and between 32% and 95% are adherent based on a systematic review of cardiovascular medications (predominantly also hypertension) (Akeroyd et al., 2015). These differences are likely due to significant variation in methodological factors and study populations. For other conditions, similar low adherence rates are found from single studies, with 47% adherence to anti-depressant medication based on a randomised control trial (RCT) (Pillai et al., 2021) and 48.1% adherence to COPD medications at baseline in another RCT (Abdulsalim et al., 2017). Given the escalating healthcare and financial burden from these non-communicable chronic in countries like India, understanding the factors which influence low and variable adherence from representative survey data is vital to guide and design interventions towards improved adherence (Dandona et al., 2017; Arokiasamy, 2018).

A conceptual model illustrating the barriers to adherence for chronic diseases highlights five core factors: patient factors, socioeconomic factors, healthcare system factors, medication factors and condition-related factors (Peh et al., 2021). Specifically in India, key barriers to adherence across these five domains include patient factors such as poor knowledge of disease and treatment; forgetfulness and preference to alternative systems of medicine (Krishnamoorthy et al., 2022); healthcare system factors such as poor doctor-patient relationships, inadequate risk communication regarding adherence, and medicine accessibility (Krishnamoorthy et al., 2022); medication factors such as side effects, regimen complexity, medication affordability and acceptability ((Raja et al., 2021; Shani et al., 2021; Krishnamoorthy et al., 2022)); socioeconomic factors such as wealth, alcohol use and disease stigma (Verma et al., 2018; Krishnamoorthy et al., 2022); and condition-related factors such as symptomatology, co-morbidities and development of complications (Choudhary et al., 2016; Aggarwal et al., 2021). Furthermore, multimorbid patients are at particular risk of poor-adherence and concomitant adverse outcomes from a range of NCDs, with disease burden and associated polypharmacy interacting to compound these barriers to adherence (Walsh et al., 2020; Foley et al., 2021; Pasina et al., 2022). Depression as a co-morbidity is of particular interest due to its prevalence in LMICs, bi-directional association with NCDs, and negative effects adherence and health outcomes (Mendenhall et al., 2014; Mensah and Collins, 2015; Udedi et al., 2018).

A thorough understanding of the barriers surrounding medication adherence is crucial. It allows the identification of patients at particular risk of poor adherence as well as the factors which may be associated with reduced adherence. Thus, it can inform public health measures that can be specifically designed to target barriers to adherence in at risk patient groups (Gast and Mathes, 2019).

Despite the availability of data on medication adherence for non-communicable chronic diseases, most studies based in India are single-centred, tend to have relatively small sample sizes, and focus on individual health conditions (Hegde et al., 2016; Nielsen et al., 2017; Yuvaraj et al., 2019; Azharuddin et al., 2021). Furthermore, most of these studies were conducted in Southern Indian states which have comparatively more efficient health systems (Rao, 2011). As such, their findings may not be generalizable to wider populations or representative at a national level. There is a lack of nation-wide analysis from large survey data to identify the socioeconomic and health-related determinants of adherence across NCDs.

This study’s primary objective was to determine the factors associated with medication adherence in patients with NCDs in India. To this end, we conducted secondary data analysis on the second wave of WHO’s Study on global AGEing and adult health (SAGE), a survey that collected data from predominantly older adults in India. We describe which socioeconomic, geographical, health-related and social-support variables are linked with the initiation and persistence components of adherence, hypothesising that lower socioeconomic status, reduced access to healthcare infrastructure, the presence of various co-morbidities and worse social support would predict poorer medication adherence in line with barriers to adherence for NCDs described in the literature (Peh et al., 2021). The findings of this study aim to inform future public health policies that improve access and adherence to medications for NCD management.

To provide further specific information for policymakers, we conducted analysis into the factors influencing adherence for the subgroup of patients who were diagnosed with each NCD of interest, the subgroup with only one NCD and the subgroup who were multimorbid (defined as the co-occurrence of two or more chronic conditions in the same individual where no single condition holds priority over the others (Nicholson et al., 2019).

## Materials and methods

### Study design, data sample and participants

We conducted a cross-sectional study, performing secondary data analysis on data from SAGE2 in India, the second wave of a survey-based global study investigating global and adult health (SAGE) from predominantly older adults (World Health Organisation, 2022b).

The STROBE checklist for observational, cross-sectional studies was utilised to inform the reporting of this study and the corresponding checklist can be found as additional file 6 (Vandenbroucke et al., 2007).

The SAGE survey was conducted in multiple countries, with the goal of using validated, standardised methods to collect community-based data from low- and middle-income countries (LMICs) to develop evidence based polices - our data analysis is from the second wave of the survey conducted in India. Technique. In India, SAGE Wave 2 was collected in 2015 through standardised survey instruments performed by appropriately trained health investigators, utilising the same multistage cluster sample design as SAGE wave 1.

The same PSUs and households covered by the 2007 SAGE wave 1 survey made up the follow-up sample for SAGE wave 2 in India. To obtain a sample of older individuals representative of India’s population, the population clusters for SAGE’s sampling were based on both geographical region and age: Sampling was undertaken in six selected states (Assam, Karnataka, Maharashtra, Rajasthan, Uttar Pradesh and West Bengal) that were selected to represent a range of geographies (north, central, east northeast, west and south) and developmental categories (based on infant mortality, female literacy, percentage of safe deliveries and *per capita* income) with one state per region and developmental category selected, as well as include a mix of rural and urban areas, to obtain a systematic random sample of individuals representative of India’s population (Arokiasamy et al., 2020; World Health Organisation, 2022b). Subsequently, the multistage cluster sampling method was utilised for selection of study participants, having two-stages in rural areas (villages and households) and in three-stages in urban areas (wards, census enumeration blocks, and households). In rural areas villages were the primary sampling units (PSUs) and in urban areas city wards were the PSUs. In rural areas in all 6 states, villages were grouped into three categories based on size (<250 households, 250–500 households, >500 households) and PSUs were selected probabilistically depending on their size. Then households were selected from each PSU using systematic random sampling while individuals within the households were selected using Kish grid tables, ensuring all age groups above 18 and both sexes were represented. In urban areas, urban wards in each state were arranged according to their size and region with PSUs selected again probabilistically based on size, then census enumeration blocks were selected randomly from each PSU and finally the households were selected using the systematic random sampling method with individuals identified via Kish tables in the same way as in rural areas. Additional file 7 provides an overview of multistage cluster sampling strategy used for in the SAGE wave 2 survey. (Arokiasamy et al., 2013; Arokiasamy et al., 2020; World Health Organisation, 2022b).

Participants of SAGE2 were predominantly over 50 years of age with a smaller comparison group of younger adult population. To achieve this, households in selected sampling units were categorised based on the age of inhabitants, with stipulated conditions for selection to interview in order to obtain the desired age distribution (Arokiasamy et al., 2020). The sampling size, coverage and scope was designed by the SAGE2 authors to result in a nationally representative selection of individuals.

SAGE Wave 2 included 9,116 completed interviews with individuals from 8,152 households: 1998 with those aged 18–49 (1,165 women and 822 men) and 7,118 with those aged over 50 (3,781 women and 3,337 men). The individual sample from each state was as follows: assam (n = 1,020), Karnataka (n = 1,095), Maharashtra (n = 1,520), Rajasthan (n = 1816), Uttar Pradesh (n = 1862) and West Bengal (n = 1803). 6,560 of respondents older than 50 were followed up from those sampled in 2007 SAGE wave 1 (these respondents were originally selected using the sample cluster-based sampling method described above). The new respondents for SAGE Wave 2 were recruited based on the same methodology and PSUs as SAGE Wave 1 to obtain an adequate sample size and avoid biases intrinsic to longitudinal surveys. Additional respondents over 50 were ‘aged-in’ from those who were 42–49 in SAGE Wave 1, additional younger individuals were surveyed from surplus younger households from Wave 1 and some households selected by Wave 1 which were unable to be interviewed at the time of this prior survey were included in Wave 2. When PSUs from SAGE Wave 1 were not accessible or traceable, no replacement was found - only PSUs from wave 1 were used. The overall response rate for the SAGE Wave 2 questionnaire 94.74% for household level responses and 77.14% for individual level responses. (Arokiasamy et al., 2020).

SAGE was developed by the WHO Evidence, Measurement and Analysis unit, based on the 2002–2004 World Health Survey. Adaptations were made based on a review of other major ageing surveys and field experts, along with further country-specific modifications. The process for country specific adaptations and translations of the standardised questionnaire followed the procedures developed and utilised for the World Health Survey. The psychometric components of the SAGE survey were reviewed and revised for SAGE2. The SAGE2 survey was first piloted through the first 100 interviews and materials were re-reviewed prior to the remainder of the survey’s implementation. SAGE2 in India used household, individual and proxy questionnaires that covered participant’s socioeconomic, health, social and cultural background as well as biomarker measures (Arokiasamy et al., 2020; World Health Organisation, 2023). The full survey materials are available online (International Institute for Population Sciences, 2023). Sub-studies of SAGE2’s components have and are being conducted, validating the metrics used by the survey (Miret et al., 2012; Snodgrass et al., 2016; World Health Organization, 2023).

SAGE2 was selected for analysis over other data sets (International Institute for Population Sciences, 2022) owing to its focus on the older adult population and extensive coverage of individuals with NCDs in India with information collection on drug access and adherence. As the same data was collected in five other LMICs, our methods and results are likely to be generalisable and comparable (Arokiasamy et al., 2020; World Health Organisation, 2022b).

### Outcomes of interest

Our primary outcome of interest was medication adherence, specifically the initiation and persistence components of adherence given the data gathered by SAGE2. Medication adherence was calculated based on those who reported having a chronic condition in the SAGE2 questionnaire and answered affirmatively to the binary question whether they took medication or treatment in the previous 2 weeks, defined by SAGE2 as those ‘currently treated’ (Arokiasamy et al., 2020). The question was precisely worded:

“Have you been taking any medications or other treatment for it *(specific disease/ condition)* in the last two weeks? (World Health Organisation, 2022b)

Adherence was calculated as the percentage of patients confirming that they took medication or other treatment for all their diagnosed chronic conditions in the last 2 weeks. Consequently, this measure and question from SAGE-2 captures adherence, according to the standard taxonomy, relating to initiation and the absence of discontinuation (persistence) of treatment. This is henceforth referred to as ‘adherence’ for simplicity although it does not capture the implementation component of adherence according to the standard taxonomy (Vrijens et al., 2012).

In the case of participants who reported depression as their NCD, this adherence question also included those attending therapy or counselling sessions as potential treatment options. In the case of participants who reported hypertension as their NCD, this adherence question also included weight loss programmes or change in eating habits as potential treatment options. For those who reported diabetes as their NCD, this adherence question specified the intake of insulin or other blood sugar lowering medications as the ‘medications or other treatment’. And for those who reported chronic lung disease as their NCD, this adherence question included the use of other treatments such as oxygen.

The following chronic conditions were included in this study: stroke, angina pectoris, diabetes mellitus, asthma, depression, hypertension and chronic lung disease. For all these conditions, long-term medication use is the standard of care, even if non-pharmacological adjuncts may also be used in some cases. Hence a negative answer to SAGE2’s treatment question predominantly implies a failure of adherence (initiation or persistence components) to pharmacological therapy—which may relate to patient, socioeconomic, healthcare, medication or condition factors under investigation. Individuals with one or more of these chronic conditions were included in analysis.

Arthritis, mouth disease, and cataracts were additional single chronic conditions assessed by SAGE2 excluded in this analysis. In these conditions, chronic medication use is not the standard of care, so data on whether patients reported taking medication or treatment in the previous 2 weeks would not be informative of what factors associated with medication adherence.

SAGE2 also asked participants whether they had been taking any medications or treatment in the previous 12 months—which was considered ‘unmet need’ by SAGE2, rather than those ‘currently treated’ (Arokiasamy et al., 2020). Additionally, patients’ memory of their behaviour over the course of a year is likely to be less accurate than their memory of recent behaviour, so this question was not considered as a proxy for medication adherence in this study. This question was used to calculate the proportion of patients who had reported taking medication in the last 12 months. To estimate the proportion of individuals who have initiated treatment but have since discontinued it, the proportion who answered affirmatively to taking medication in the last 12 months and also reported non-adherence over the last 2 weeks was determined.

### Explanatory variables

Potential explanatory variables for this study’s primary outcome were categorised into demographic, socioeconomic, health-related, social support and geographical variables. The choice of these were informed by a review of key literature (Lee et al., 2020; Chauhan et al., 2022) which used similar data sources to explore the factors underpinning health-outcomes with multifactorial contributing factors.

Data on household level factors such as wealth, location, age, and sex were taken from the household-level questionnaire part of SAGE2.

Most outcome measures were taken from the individual level questionnaires performed as part of SAGE2. Additional file 1 shows the exact questions used in the SAGE questionnaire and measurement details. Health related explanatory variables used included subjective measures of anxiety and depression rather than clinical diagnoses of these conditions, as a clinical depression was one of the NCDs of interest.

Relevant categories that had to be excluded were parental education (owing to a response rate of ∼25% with responses subdivided into a large number of distinct categories) and whether participants had health insurance (as very few participants responded) (Arokiasamy et al., 2020).

### Bias

Potential sampling bias was mitigated through the use of post-stratified weights in analysis. SAGE2 calculated household and individual weights for analysis, determined based on the probability of selection at every stage. Weights were post-stratified to reduce sampling error and non-response bias. These weights were used for all this study’s analyses as stipulated in the SAGE2 report in order to ensure the generalisability of results (Arokiasamy et al., 2020).

The use of weights only allows the calculation of proportions of respondents adhering for explanatory variables of interest—additional file 5 uses unweighted data to provide the raw numbers, as well as proportions, of participants for each explanatory variable for reference (unweighted data was not used in any analysis in this paper).

### Data analysis

#### Data processing

The original raw dataset was processed using R for all analyses in this study (R Core Team, 2022).

Nine columns were missing labels after ingestion and so the correct labels were assigned based on the original questionnaire.

#### Sample characteristics determined and summary metrics computed

The sample’s base characteristics were determined, and summary metrics were computed. Responses rates were generally >99% on included questions, with the exceptions of alcohol use (13% response rate), working status (50%), community support (15%), healthcare provider (69%) and highest level of schooling (59%). (See additional Table 2 for response rates by question).

All summary metrics and downstream statistics were determined based on available data, utilising sample weights as stipulated, to reduce any bias arising from missing data and avoid use of imputation.

#### Bivariate analysis

To compare adherent and non-adherent groups, bivariate analysis was undertaken.

For discrete variables the significance level was calculated using the chi-square test, whilst for the single continuous variable (number of chronic conditions), the Welch Two Sample *t*-test was used. Both tests were calculated on data weighted using the individual weights from the SAGE2 data. 95% confidence intervals were calculated based on the Wald type from the data, with statistics informed by Lumley and Scott’s work on regression analysis of complex weighted survey data (Lumley and Scott, 2017).

Table 1 details groupings utilised in bivariate analysis to facilitate more meaningful comparisons.

**TABLE 1:** Variables and groupings used in Bivariate Analysis. Table shows domain analysed, how the responses from the SAGE-2 dataset were grouped and why responses were grouped in this way. The exact questions and response options used in the SAGE-2 survey can be found in additional file 1.

Question	Grouping	Justification
Age	<50 and ≥50	This grouping of age is the same used in the SAGE2 analysis and allows more meaningful comparisons within a dataset where sampling focused on older adults (≥50)
Education	**Less than primary school/do not know** (No formal education, Less than primary school,Don’t know)	Proportion of participants with significantly higher education was low and it was unlikely to significantly affect medication adherence in these settings
	**Secondary school and below** (Primary school completed, Secondary school completed)	
	**Past secondary school** (High school (or equivalent) completed, College/pre-university/university completed,Post-graduate completed)	
Religion	**Hinduism**, **Islam**, **Other** (*including* Buddhism, Chinese traditional religion, Christianity, Jainism, Judaism, Primal indigenous, Sikhism, Others, No/None and Refused)	Fewer than 5% of people were in the categories grouped under other
Primary Healthcare Provider	**Private** (Private doctor’s office, Private clinic/Healthcare facility, Private hospitals')	
	**Public** (Public clinic/Healthcare facility, Public hospital)	
	**Community** (Charity/Church run clinic, Charity/Church run hospital, Traditional healer, Pharmacy/Dispensary)	
	**Other** (Others)	
Marital Status	**Currently Married**	
	**Not Currently Married** (widowed, separated/divorced, cohabiting, never married)	
Caste	**Schedule Tribe**	Schedule Caste and Schedule Tribe are different socially marginalised groups facing different challenges. Other groups were combined as they are relatively privileged
	**Schedule Caste**	
	**Other** (Other Backward Caste, None of the Above, Others, Don’t Know)	
Likert Scales: Difficulty Moving, Difficulty Remembering, Feeling Low, Feeling Anxious	**Moderate or worse** (*including moderate, severe, extreme/cannot do*)	
	**None/Mild** (*including none, mild*)	
Likert Scale: Health Today	**Poor** (*including* moderate, bad and very bad health today)	
	**Good** (*including* good and very good health today)	

#### Multivariate logistic regression modelling

Multivariate logistic regression was used to interrogate the key variables underlying medication adherence. Adherence was used as the outcome variable and key explanatory variables were used based on bivariate analysis where there was a statistically significant relationship (*p* < 0.05) with adherence.

The following variables that achieved significance in bivariate analysis were excluded from multivariate analysis: working status (as over 50% of respondents failed to answer), region and caste (very broad qualitative variables which would limit generalisability of findings as they are highly specific to India).

Thus the following covariates were used in multivariate modelling: wealth quintile, age, number of chronic conditions, rural living, a subjective measure of feelings of anxiety, a subjective measure of feelings of depression, a subjective measure of cognition, tobacco use, educational status. Every field used for multivariate modelling had <1% non-responders.

Variable selection was performed the maximise the strength of the model based on the AIC metric—all variables that achieved significance in bivariate modelling increased the strength of the model except for the subjective measure of cognitive memory and thus it was not included in the final model.

To check for multicollinearity, the generalised Variance Inflation Factors were calculated for the covariates used in the model (Fox and Monette, 1992).

Forest plots were produced to depict the odds ratios of covariates.

All code used for analysis can be found in data availability section below.

#### Subgroup analysis

Analysis of factors influencing adherence in certain subsets of the population was also conducted.

1. The factors influencing adherence for multimorbid individuals
a. Multimorbidity was defined as the simultaneous presence of two or more NCDs of interest in a single individual.
2. The factors influencing adherence for individuals with only one morbidity
3. The factors influencing adherence for each NCD of interest

Only bivariate analysis was conducted for these subsets of individuals. Further logistic regression analysis was not performed for these subgroups with more limited sample sizes.

### Ethics

The SAGE study (Arokiasamy, 2013; Arokiasamy et al., 2020; World Health Organisation, 2022b) was conducted according to the guidelines laid down in the Declaration of Helsinki and all procedures involving human subjects/patients were approved by the World Health Organization’s Ethical Review Board, Geneva and the Institutional Review Board, International Institute of Population Sciences, Mumbai, India. The survey agencies that conducted the field survey for the data collection have collected prior written consent from all respondents. This dataset provided is anonymized and does include any private or sensitive information of the participants which can be used to identify them.

The International Institute for Population Studies also provided approval to conduct this study.

## Results

### Sample characteristics

The study sample consisted of 9,116 participants from India, of which 2,840 were diagnosed with one or more NCDs relevant in this study. Of these, 2037 only had one morbidity while 803 had two or more morbidities (multimorbid). Full demographic details across domains assessed by the SAGE2 survey were calculated as part of the original study can be found in their full report (Arokiasamy et al., 2020).

The overall rate of medication adherence was 51% and Table 2 depicts how this varies by NCD.

**TABLE 2:** weighted adherence rates from SAGE2 dataset by morbidity status and disease status.

NCD	Non-adherent (%)	Adherent (%)
Any Morbidity	48.62	51.38
Multimorbid	62.48	37.52
Single morbidity only	43.20	56.80
Angina	36.94	63.06
Diabetes	35.89	64.11
Asthma	42.69	57.31
Hypertension	42.37	57.63
Stroke	46.60	53.40
Lung Disease	56.77	43.23
Depression	66.81	33.19

### Results of bivariate analysis

Table 3 shows how each potential explanatory variable is associated with the rate of adherence for all 2,840 individuals with one or more morbidity. The reference category was the default baseline from survey data.

**TABLE 3:** Factors associated with medication adherence in patients with one or more morbidities, n = 2,840.

Characteristic	Non-adhering	Adhering	*p*-value
**Age (in years)**	----	----	<0.001
<50	16.62%	10.53%	--
≥50	83.38%	89.47%	--
**Sex**	----	----	0.426
Male	45.68%	47.44%	--
Female	54.32%	52.56%	--
**Wealth quintile**	----	----	0.008
1 (poorest 20%)	17.59%	12.48%	--
2	17.54%	13.92%	--
3	15.73%	18.90%	--
4	21.53%	21.72%	--
5 (wealthiest 20%)	27.61%	32.99%	--
**Number of Chronic Conditions [mean (SD)]**	1.5 (0.74)	1.2 (0.5)	<0.001
**Place of residence**	----	----	0.006
Urban	27.02%	37.10%	--
Rural	72.98%	62.90%	--
**Region**	----	----	<0.001
Assam	11.71%	4.10%	--
Karnataka	9.15%	17.36%	--
Maharashtra	19.21%	17.72%	--
Rajasthan	19.85%	12.04%	--
Uttar Pradesh	21.59%	21.65%	--
West Bengal	18.49%	27.14%	--
**Marital Status**	----	----	0.404
Currently Married	74.24%	75.87%	--
Not Currently Married	25.76%	24.13%	--
**Religion**	----	----	0.231
Hinduism	82.31%	84.43%	--
Islam	12.96%	12.49%	--
Other/None	4.73%	3.07%	--
**Ethnic Group**	----	----	0.002
Schedule Tribe	6.33%	4.07%	--
Schedule Caste	16.42%	12.50%	--
Others	77.25%	83.43%	--
**Ever smoked**	----	----	0.021
No	62.40%	68.79%	--
Yes	37.60%	31.21%	--
**Consumed alcohol last 30 days**	----	----	0.344
Yes	51.02%	44.75%	--
No	48.98%	55.25%	--
**Rate your health**	----	----	0.485
Poor	68.67%	70.42%	--
Good	31.33%	29.58%	--
**Difficulty in cognition with concentrating/remembering things**	----	----	0.036
Moderate or worse	38.42%	32.84%	--
None/Mild	61.58%	67.16%	--
**Difficulty in moving around**	----	----	0.397
Moderate or worse	41.59%	39.42%	--
None/Mild	58.41%	60.58%	--
**Community Support**	----	----	0.152
Yes	39.03%	48.19%	--
No	60.97%	51.81%	--
**Currently working**	----	----	0.008
Yes	60.63%	50.50%	--
No	39.37%	49.50%	--
**Healthcare provider**	----	----	0.214
Private	66.40%	69.62%	--
Public	26.39%	22.67%	--
Community	3.92%	5.25%	--
Other	3.29%	2.46%	--
**Ever Been to School**	----	----	<0.001
Yes	58.65%	66.27%	--
No	41.35%	33.73%	--
**Level of Education**	----	----	0.315
Less than primary school/do not know	23.16%	23.72%	--
Secondary school and below	48.12%	43.45%	--
Past secondary school	28.73%	32.83%	--
**Feeling sad/low/depression**	----	----	0.012
None/Mild	69.97%	75.24%	--
Moderate or worse	30.03%	24.76%	--
**Worry or anxiety**	----	----	0.005
None/Mild	57.03%	64.53%	--
Moderate or worse	42.97%	35.47%	--
**Cataracts**	----	----	0.831
No	76.05%	75.53%	--
Yes	23.95%	24.47%	--
**Money to meet needs**	----	----	0.795
Completely	8.51%	9.53%	--
Mostly	15.64%	17.61%	--
Moderately	45.56%	44.17%	--
A little	22.91%	21.83%	--
None at all	7.39%	6.86%	--

Abbreviations: SD, standard deviation.

Sociodemographic variables significantly associated with adherence were age group with older people more likely to adhere (*p* < 0.001), rural-urban living with those living rurally less likely to adhere (*p* = 0.006), state with Assam showing particularly low adherence and West Bengal particularly high adherence (*p* < 0.001), ethnic group with those from Scheduled Caste and Scheduled Tribe groups less likely to adhere (*p* = 0.002), schooling status with those who had never been to school less likely to adhere (*p* < 0.001). Sex (*p* = 0.426), religion (*p* = 0.231) and level of education (*p* = 0.315) was not significantly associated with adherence.

Economic variables significantly associated with adherence were wealth quintile (*p* = 0.008), with wealthier individuals more likely to adhere, and working status, with those currently working less likely to adhere (*p* = 0.008). Healthcare provider (*p* = 0.214) and whether participants felt they had enough money to meet their needs (*p* = 0.795) were not significantly associated with adherence.

Health related variables significantly associated with adherence were multimorbidity (*p* < 0.001), smoking with smokers less likely to adhere (*p* = 0.021), impaired cognition with those reporting difficulty concentrating less likely to adhere (*p* = 0.036), anxiety with anxious individuals less likely to adhere (*p* = 0.005) and depression with those feeling low or depressed less likely to adhere (*p* = 0.012). Self-rated health (*p* = 0.485), presence of cataracts (*p* = 0.175) and difficulty moving around (*p* = 0.397) were not significantly associated with adherence.

Community related factors such as community support (*p* = 0.152) and marital status (*p* = 0.404) did not have a statistically significant association with adherence.

Subgroup analysis - Multimorbidity and Single Morbidity: Factors associated with medication adherence.

Table 4 reports the bivariate analysis for the subgroup of individuals who were multimorbid with two or more chronic conditions. Only age, with older individuals being more likely to adhere (*p* < 0.001), and region (*p* < 0.001) were significantly associated with adherence.

**TABLE 4:** Factors associated with medication adherence in patients with 2 or more chronic conditions (n = 803).

Characteristic	Non-adherent	Adherent	*p*-value
**Age (in years)**	----	----	<0.001
<50	9.78%	2.59%	--
≥50	90.22%	97.41%	--
**Sex**	----	----	0.209
Male	48.89%	54.19%	--
Female	51.11%	45.81%	--
**Wealth quintile**	----	----	0.337
1 (poorest 20%)	13.83%	8.92%	--
2	13.95%	13.14%	--
3	14.87%	17.20%	--
4	17.69%	20.49%	--
5 (wealthiest 20%)	39.66%	40.24%	--
**Place of residence**	----	----	0.455
Urban	36.84%	41.04%	--
Rural	63.16%	58.96%	--
**Region**	----	----	<0.001
Assam	11.05%	2%	--
Karnataka	10.76%	21.09%	--
Maharashtra	18.05%	18.90%	--
Rajasthan	18.94%	14.20%	--
Uttar Pradesh	20.39%	13.20%	--
West Bengal	20.82%	30.61%	--
**Marital Status**	----	----	0.69
Currently Married	74.06%	75.55%	--
Not Currently Married	25.94%	24.45%	--
**Religion**	----	----	0.198
Hinduism	79.11%	83.75%	--
Islam	15.20%	14.18%	--
Other/None	5.68%	2.07%	--
**Ethnic Group**	----	----	0.162
Schedule Tribe	4.51%	3.81%	--
Schedule Caste	15.74%	9.84%	--
Others	79.75%	86.35%	--
**Ever smoked**	----	----	0.91
No	66.18%	66.63%	--
Yes	33.82%	33.37%	--
**Consumed alcohol last 30 days**	----	----	0.988
Yes	44.09%	43.88%	--
No	55.91%	56.12%	--
**Rate your health**	----	----	0.736
Poor	73.23%	74.60%	--
Good	26.77%	25.40%	--
**Difficulty in cognition with concentrating/remembering things**	----	----	0.111
Moderate or worse	43.38%	35.90%	--
None/Mild	56.62%	64.10%	--
**Difficulty in moving around**	----	----	0.678
Moderate or worse	47.78%	49.53%	--
None/Mild	52.22%	50.47%	--
**Community Support**	----	----	0.578
Yes	48.97%	55.19%	--
No	51.03%	44.81%	--
**Currently working**	----	----	0.06
Yes	48.55%	36.49%	--
No	51.45%	63.51%	--
**Healthcare provider**	----	----	0.273
Private	70.41%	76.21%	--
Public	23.07%	20.17%	--
Community	4.79%	3.35%	--
Other	1.73%	0.27%	--
**Ever Been to School**	----	----	0.709
Yes	68.43%	70%	--
No	31.57%	30%	--
**Level of Education**	----	----	0.212
Less than primary school/do not know	23.23%	25.01%	--
Secondary school and below	42.94%	35.17%	--
Past secondary school	33.83%	39.81%	--
**Feeling sad/low/depression**	----	----	0.206
None/Mild	69.67%	74.60%	--
Moderate or worse	30.33%	25.40%	--
**Worry or anxiety**	----	----	0.317
None/Mild	56.74%	61.63%	--
Moderate or worse	43.26%	38.37%	--
**Cataracts**	----	----	0.904
No	66.81%	67.41%	--
Yes	33.19%	32.59%	--
**Money to meet needs**	----	----	0.945
Completely	10.18%	10.51%	--
Mostly	19.08%	17.12%	--
Moderately	41.61%	44.17%	--
A little	23.46%	21.67%	--
None at all	5.67%	6.53%	--

Abbreviations: SD, standard deviation.

Table 5 shows bivariate analysis for the subgroup of individuals who only had one chronic morbidity. Akin to the general morbidity analysis age (*p* < 0.001), region (*p* < 0.001), wealth quintile (*p* = 0.003), rural-urban living (*p* < 0.001), region (*p* < 0.001), ethnic group (*p* = 0.003), working status (*p* = 0.004), having been to school (*p* < 0.001), smoking status (*p* = 0.013), depression (*p* = 0.045), anxiety (*p* = 0.01) were significant predictors of medication adherence. However, in contrast to general morbidity analysis community support (*p* = 0.049) was also significantly associated with adherence. In the population with only one morbidity, difficulty concentrating (*p* = 0.196) was not significant unlike the general morbid population.

**TABLE 5:** Factors associated with medication adherence in patients only 1 chronic condition (n = 2037).

Characteristic	Non-adherent	Adherent	*p*-value
**Age (in years)**	----	----	<0.001
<50	20.49%	12.58%	--
≥50	79.51%	87.42%	--
**Sex**	----	----	0.477
Male	43.87%	45.70%	--
Female	56.13%	54.30%	--
**Wealth quintile**	----	----	0.003
1 (poorest 20%)	19.72%	13.40%	--
2	19.57%	14.12%	--
3	16.21%	19.33%	--
4	23.70%	22.03%	--
5 (wealthiest 20%)	20.79%	31.11%	--
**Place of residence**	----	----	<0.001
Urban	21.47%	36.09%	--
Rural	78.53%	63.91%	--
**Region**	----	----	<0.001
Assam	12.09%	4.64%	--
Karnataka	8.24%	16.40%	--
Maharashtra	19.87%	17.41%	--
Rajasthan	20.36%	11.48%	--
Uttar Pradesh	22.27%	23.83%	--
West Bengal	17.18%	26.24%	--
**Marital Status**	----	----	0.432
Currently Married	74.34%	75.95%	--
Not Currently Married	25.66%	24.05%	--
**Religion**	----	----	0.683
Hinduism	84.12%	84.61%	--
Islam	11.69%	12.06%	--
Other/None	4.19%	3.33%	--
**Ethnic Group**	----	----	0.003
Schedule Tribe	7.36%	4.14%	--
Schedule Caste	16.81%	13.18%	--
Others	75.83%	82.68%	--
**Ever smoked**	----	----	0.013
No	60.27%	69.35%	--
Yes	39.73%	30.65%	--
**Consumed alcohol last 30 days**	----	----	0.162
Yes	54.43%	45.04%	--
No	45.57%	54.96%	--
**Rate your health**	----	----	0.263
Poor	66.08%	69.34%	--
Good	33.92%	30.66%	--
**Difficulty in cognition with concentrating/remembering things**	----	----	0.196
Moderate or worse	35.62%	32.05%	--
None/Mild	64.38%	67.95%	--
**Difficulty in moving around**	----	----	0.68
Moderate or worse	38.08%	36.81%	--
None/Mild	61.92%	63.19%	--
**Community Support**	----	----	0.049
Yes	31.93%	45.62%	--
No	68.07%	54.38%	--
**Currently working**	----	----	0.004
Yes	66.81%	54.64%	--
No	33.19%	45.36%	--
**Healthcare provider**	----	----	0.093
Private	63.98%	67.63%	--
Public	28.39%	23.42%	--
Community	3.40%	5.83%	--
Other	4.23%	3.12%	--
**Ever Been to School**	----	----	<0.001
Yes	53.12%	65.30%	--
No	46.88%	34.70%	--
**Level of Education**	----	----	0.232
Less than primary school/do not know	23.11%	23.36%	--
Secondary school and below	51.89%	45.74%	--
Past secondary school	25%	30.90%	--
**Feeling sad/low/depression**	----	----	0.045
None/Mild	70.14%	75.41%	--
Moderate or worse	29.86%	24.59%	--
**Worry or anxiety**	----	----	0.01
None/Mild	57.19%	65.28%	--
Moderate or worse	42.81%	34.72%	--
**Cataracts**	----	----	0.175
No	81.29%	77.62%	--
Yes	18.71%	22.38%	--
**Money to meet needs**	----	----	0.407
Completely	7.56%	9.27%	--
Mostly	13.70%	17.74%	--
Moderately	47.78%	44.17%	--
A little	22.60%	21.87%	--
None at all	8.36%	6.95%	--

Abbreviations: SD, standard deviation.

### By disease: Factors associated with medication adherence

We report the bivariate analysis for the subgroup of individuals for each chronic condition (see additional file 4).

Factors which have statistically significant association with adherence are generally consistent across diseases but due to reduction of the effective sample size, fewer variables achieved significance at the *p* < 0.05 threshold.

Adherence to asthma medication was found to be associated with age, with older individuals more likely to adhere (*p* = 0.025) and working status, with those currently not working more likely to adhere (*p* = 0.024). Adherence to diabetes medication was also associated with age, with older individuals more likely to adhere (*p* = 0.04); wealth quintile, with wealthier individuals more likely to adhere (*p* = 0.09); region, with those in Assam and Rajasthan being most likely not to adhere (*p* < 0.001); ethnic group, with those in Schedule Caste and Schedule Tribe groups being less likely to adhere (*p* = 0.003); difficulty concentrating with those reporting difficulty concentrating being less likely to adhere (*p* < 0.001); feeling low or depressed (*p* < 0.001) and symptoms of anxiety (*p* = 0.002) were also associated with non-adherence to diabetes medication. Adherence to hypertension medication was associated with age, with those older found to be more likely to adhere (*p* = 0.002); ethnic group, with those in Schedule Caste or Schedule Tribe groups being more likely not to adhere (*p* = 0.003); self-ratings of health; with those in subjective poor health being more likely to adhere (*p* = 0.024); working status, with those currently working being less likely to adhere (*p* = 0.009); schooling status, with those who have been to school being more likely to adhere (*p* = 0.011). Adherence to other lung disease medication was found to be associated with region, with those in Assam, Uttar Pradesh and Rajasthan being most likely to not adhere (*p* = 0.005) and feelings of worry or anxiety, with those feeling anxious being less likely to adhere (*p* = 0.044). Adherence to stroke medication was associated with age, with those older found to be more likely to adhere (*p* = 0.004) and feelings of worry or anxiety, with those feeling anxious being more likely to adhere (*p* = 0.017). Adherence to depression medication was not found to be associated with any factors.

### Results of multivariate analysis

**FIGURE 1:**
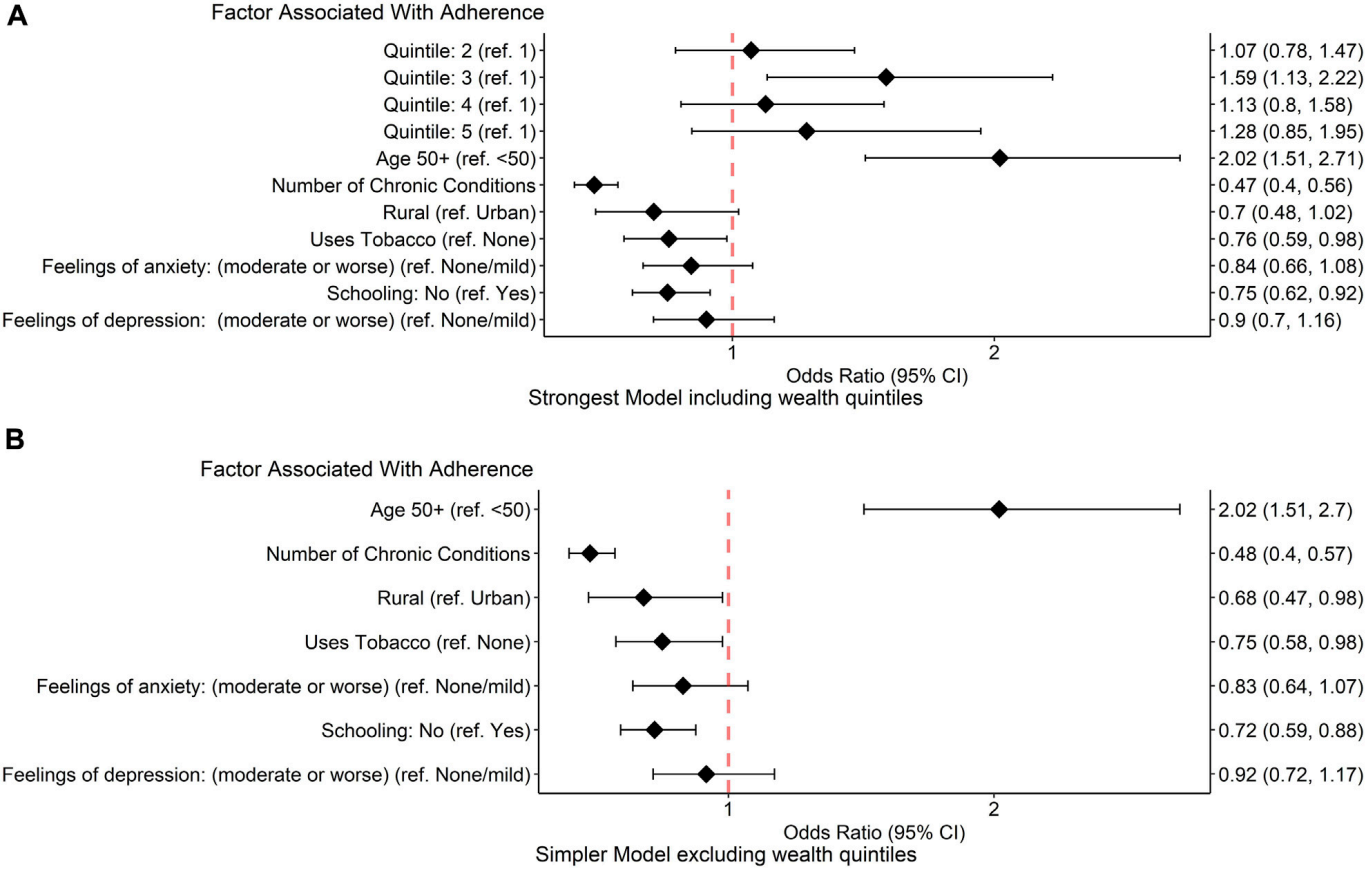
Multivariate logistic regression analysis. The forest plots show the results of multivariate logistic regression analysis. **(A)**: shows the strongest model including wealth quintile (wealth quintile 1 represents the poorest 20% and wealth quintile 5 represents the wealthiest 20%). **(B)** Shows a simpler model without wealth quintile. Odds ratios (diamonds) with 95% confidence intervals (error bars) for factors associated with medication adherence are also displayed to the right of the figure. Higher odds ratios represent increased odds of adherence. Abbreviations: CI, confidence interval, ref, reference.

Table 6; Figure 1 show the results of multivariate logistic regression model. The generalised collinearity test showed no evidence of multicollinearity, with all variables below 1.5, indicating they are thus independently associated with medication adherence.

**TABLE 6:** Multivariate analysis showing factors associated with medication adherence. Table 6a shows the strongest model including wealth quintile (wealth quintile 1 represents the poorest 20% and wealth quintile 5 represents the wealthiest 20%). Table 6b shows a simpler model without wealth quintile.

Variable	aOR	95% CI (LL, UL)	*p*-value
(a)			
Age 50+ (ref. <50)	2.02	(1.51, 2.7)	<0.001
Number of Chronic Conditions	0.48	(0.4, 0.57)	<0.001
Rural (ref. Urban)	0.68	(0.47, 0.98)	0.039
Uses Tobacco (ref. None)	0.75	(0.58, 0.98)	0.035
Feelings of anxiety: (moderate or worse) (ref. None/mild)	0.83	(0.64, 1.07)	0.156
Schooling: No (ref. Yes)	0.72	(0.59, 0.88)	0.002
Feelings of depression: (moderate or worse) (ref. None/mild)	0.92	(0.72, 1.17)	0.491

Variable	aOR	95% CI (LL, UL)	*p*-value
(b)			
Quintile: 2 (ref. Quintile 1)	1.07	(0.78, 1.47)	0.666
Quintile: 3 (ref. Quintile 1)	0.49	(1.13, 2.22)	0.008
Quintile: 4 (ref. Quintile 1)	1.13	(0.8, 1.58)	0.488
Quintile: 5 (ref. Quintile 1)	1.28	(0.85, 1.95)	0.242
Age 50+ (ref. <50)	2.02	(1.51, 2.71)	<0.001
Number of Chronic Conditions	0.47	(0.4, 0.56)	<0.001
Rural (ref. Urban)	0.7	(0.48, 1.02)	0.067
Uses Tobacco (ref. None)	0.76	(0.59, 0.98)	0.035
Feelings of anxiety: (moderate or worse) (ref. None/mild)	0.84	(0.66, 1.08)	0.175
Schooling: No (ref. Yes)	0.75	(0.62, 0.92)	<0.001
Feelings of depression: (moderate or worse) (ref. None/mild)	0.9	(0.7, 1.16)	0.42

aoR, adjusted odds ratio; LL, lower limit; UL, upper limit.

The strongest model (AIC = 3,995) in Figure 1A; Table 5a included wealth. However, increasing wealth was only weakly associated with improved adherence and exclusion of wealth resulted in a slight decrease in model strength (AIC = 4,004) in Figure 1A; Table 6b.

The strongest factor predicting non-adherence to medication across these components was multimorbidity (odds ratio 0.47, 95% CI 0.40–0.56). Tobacco use (0.76, 0.59–0.98) and never having attended school (0.75, 0.62–0.92) were all significantly associated with poor medication adherence (*p* < 0.05) while rural living (0.70, 0.48–1.02), feelings of anxiety (0.84, 0.66–1.08) and feelings of depression (0.9, 0.70–1.16) were factors lacking statistically significant association with medication adherence. Older age (2.02,.51–2.71) was significantly associated with improved medication adherence (*p* < 0.05) whilst there was a weak association between increased wealth and improved medication use.

### Medication-use at 12 months

Over the preceding 12 months period, 44.3% of those with 1 or more NCDs reported using all their medications or treatments as prescribed. Of those who were adherent over 2 weeks (the primary outcome measure), 97.5% also reported adherence over the previous 12 months (Table 6).

In contrast, of those who were not adherent over the previous 2 weeks, 75.0% were also not adherent over the previous 12 months while 25.0% of those were adherent over the previous 12 months (Table 7).

**TABLE 7:** adherence over previous 12 months and relationship to adherence over previous 2 weeks.

	Adherent to all medications over previous 12 months (%)	Non-adherent to all medications over previous 12 months (%)
One or more NCD	55.66	44.34
Adherent to all medications over the previous 2 weeks	97.5	2.50
Non-adherent to all mediations over previous 2 weeks	24.96	75.04

Abbreviations: NCD, non-communicable disease.

## Discussion

### Summary

Adherence to medications for NCDs is key for improving quality of life, reducing the risk of premature complications, and increasing life span. We hypothesised that a variety of socioeconomic, geographical, health-related, and social-supported variables are linked with medication adherence. In this largescale analysis of factors associated with adherence across India, we show that the strongest risk factor for the low medication adherence was a health-related factor, multimorbidity. Moreover, we find that lower wealth and educational attainment (socioeconomic characteristics), tobacco use, feelings of depression, and feeling of anxiety (health-related variables) and rural living (geographical variable) are further factors associated with patients not adhering to treatment albeit with more mixed effect sizes. Region and caste may be further India-specific variables associated with adherence, but these were not analysed in multivariate modelling as they are broad qualitative factors which may have limited generalisability of findings. Other variables interrogated were not found to be significantly associated with medication adherence in this study. Failure of medication adherence in this study reflects either non-initiation or lack of persistence of treatment.

### Comparisons with existing literature and implications

The overall medication adherence rate in this study, indicating only one in two patients take their prescribed medications, is similar to that postulated by the WHO for chronic conditions (World Hea lth Organisation, 2003) but lower than that observed in several facility based studies in India among patients with NCDs (Basu et al., 2013a; Basu et al., 2018; Shalini, 2020). This finding reiterates that patients who choose to engage with health systems will tend to report improved adherence and access, but such results cannot always be generalised to the wider situation in more vulnerable communities (Sankar et al., 2015; Balasubramanian et al., 2018) who experience the greatest challenges in accessing healthcare.

Reported use of medication over the previous 12 months was lower than over the previous 2 weeks as expected. Among those who were non-adherent to medication, the majority were also non-adherent over the previous 12 months, indicating a failure of treatment initiation or a long-term failure of persistence. 25% of individuals reported adherence over the previous 12 months but not in the previous 2 weeks, implying a recent failure of persistence to treatment. This finding is in keeping with available literature in LMICs like India, as well as in wider systematic reviews, where a combination of barriers affects the different components of adherence to NCD treatment (Kardas et al., 2013; Dalal et al., 2021; Krishnamoorthy et al., 2022). Financial non-adherence due to the depletion of medication stocks and recent financial strain may contribute further to this phenomenon (Basu et al., 2013b; Osborn et al., 2017).

Our results, showing multimorbidity halves the odds of adherence, are consistent with the growing evidence that suggests multimorbidity can reduce medication adherence (Maffoni et al., 1999; Walsh et al., 2020; Allaham et al., 2022; Pasina et al., 2022). For the multimorbid population specifically, we found that only age, region and increasing number of comorbidities were significant factors influencing adherence. Hence, multimorbidity may influence adherence through compounding patient and health-system related factors. This exacerbation of smaller individual effects may occur through each of the aforementioned five key barriers to adherence (Peh et al., 2021): patient factors, e.g., forgetfulness and competing needs worsened by multimorbidity (Peh et al., 2021); socioeconomic factors, e.g., socioeconomic status and health literacy as a corollary of low education standards (Miller, 2016); healthcare system factors, e.g., medication affordability and accessibility (Chudiak et al., 2018; Allaham et al., 2022); medication factors, e.g., regimen complexity, pooling of side effects and negative drug interactions (Pasina et al., 2022); and condition-related factors, e.g., development of complications and multimorbidity symptomatology (Choudhary et al., 2016; Aggarwal et al., 2021).

We also show that a variety of other factors independently affect medication adherence, additional to the influence of multimorbidity.

In the present study, older age participants were more likely to adhere to medication compared to the younger patients. There is conflicting evidence in this regard although meta-analyses focussing on patients with hypertension and diabetes have also found that overall older age increased odds of adherence (Nielsen et al., 2017; Azharuddin et al., 2021). Despite challenges in maintaining adherence in older adults due to cognitive decline, memory loss, and reduced visual acuity, older patients may fare better than younger patients, especially recently diagnosed individuals who may lack adequate belief in medication (Isacson and Bingefors, 2002).

This study found that low educational status tended to be associated with poor adherence. There is mixed evidence in this regard, whereby some studies have noted a relationship with poor adherence (Azharuddin et al., 2021; Chauke et al., 2022), whereas evidence from a nationally representative survey from India (2015–16) did not find an association with educational status (Singh et al., 2022). These studies focussing on LMICs have reported factors such as knowledge of medications or disease and negative perceptions about medications, to be associated with poor adherence (Bowry et al., 2011; Azharuddin et al., 2021; Chauke et al., 2022). Such factors may be correlated with lower educational status - and thus reinforce the need for adequate counselling and advice from healthcare professionals when prescribing medications.

We show that subjective feelings of depression and anxiety were predictors of non-adherence, albeit with small effect sizes and were found to be non-significant on multivariate analysis, which may be related to the use of a subjective rather than objective clinical measure. The link between depression, anxiety and poor adherence is reflected in existing literature: findings from a recent scoping review (Chauke et al., 2022) focussed on LMICs identified negative attitudes including depression as a key theme underpinning poor medication adherence, while anxiety and poor quality of life were additional emerging themes. Depression has previously been associated with poor adherence in wider meta-analyses (Gonzalez et al., 2008; D et al., 2000; Grenard et al., 2011). The combination of depression and non-adherence to medications for other non-communicable chronic diseases can have deleterious health consequences (Gonzalez et al., 2008; Poletti et al., 2022) demonstrating the need to strengthen screening and primary care support for individuals with NCDs and comorbid depression.

Unlike some previous studies from India and LMICs where female gender was associated with improved adherence to medications (Nielsen et al., 2017; Singh et al., 2022), sex was not a significant predictor of mediating non-adherence in our study. This could be because the present study participants were predominantly restricted to older age-groups. This study found a weak association between wealth and medication adherence. Although lower socioeconomic status is commonly linked with poor adherence, similar studies and reviews have also found weak associations (Gast and Mathes, 2019; Singh et al., 2022). This may be due to the way wealth was calculated as part of SAGE2 which may not truly reflect socioeconomic status (see additional file 1) as well as complex interactions between wealth and other health-system and patient factors that influence medication adherence (Tedla and Bautista, 2017; Vishnu et al., 2017; Stirratt et al., 2015; Ved et al., 2019).

### Limitations

This study has certain key limitations.

First, we could not ascertain if patients were taking all their medication doses according to prescribed instructions (implementation component of adherence) (Vrijens et al., 2012) as this was not captured by the SAGE2 survey. As a result, our findings are restricted to understanding the initiation and continued persistence of therapy. Unquantified levels of non-adherence with the implementation phase of treatment likely mean our results underestimate the prevalence of medication non-adherence. Furthermore, a minority of patients with diabetes in India may take exclusively complementary and alternative medicine (CAM), which can be ineffective in lowering blood sugar levels—this survey tool could not differentiate between use of modern medications or only CAM(73). Additionally, it is possible that a proportion of patients with depression, chronic lung disease and/or hypertension as their NCD may only have been prescribed non-pharmacological therapies (counselling, oxygen and weight loss programmes respectively) which were included by SAGE2 in their currently treated question—as a result, for these patients, their response may refer to the initiation and persistence of use of these treatment options which may have different (albeit related) factors influencing adherence. Secondly, the SAGE survey only asked whether respondents had taken medication and did not ask for the specific reasons for non-adherence. As such, there was no way to distinguish between behavioural and access-related factors for adherence. Additionally SAGE2 did not assess factors such as knowledge of medications, the patient’s knowledge of their disease, negative perceptions about medications, high medication costs or experience of side effects which have been found to be important factors associated with adherence in reviews focussed on LMICs (Bowry et al., 2011; Chauke et al., 2022)Nevertheless, to our knowledge, SAGE2 is the only large-scale survey in India to date that captured information on both initiation and persistence of medications in NCDs. An important research implication is that future, similar national surveys should differentiate the three adherence components of initiation, implementation, and discontinuation, specifically assessing whether medication was taken in line with the prescribed dosing regimen to examine the implementation component, as well as assessing a greater number of factors that may represent barriers to adherence.

Thirdly, social desirability bias is often present in adherence research when assessed through subjective questionnaires (Stirratt et al., 2015; Tedla and Bautista, 2017). Such phenomena increase the likelihood that our results overestimate the prevalence of adherence, implying that the issue of non-communicable chronic disease related medication adherence may constitute a greater public health challenge than previously envisaged.

Fourthly, as this study’s data was cross-sectional, we were unable to infer causal relations in the absence of prospective data. Although this study is based on a nationally representative sample of the Indian older adults’ population and so may be able to be generalised to older adults across the nation, information is only available on six states and the sampling methodology renders it largely unfit for generalising to the younger portion of the population. Finally, SAGE2’s sampling took place prior to major public health initiatives that improved access to high quality generic medications and strengthened primary health systems, which may have improved adherence in the general population (Ved et al., 2019).

### Implications

It is likely that the findings of this study are applicable to other LMICs, given the similarities in factors identified which are associated with adherence. Understanding the factors which affect medication adherence is key to determining cost-effective interventions to improve adherence. Such interventions have the potential to improve treatment outcomes and reduce healthcare costs (Sokol et al., 2005; Simpson et al., 2006). For example, evidence that individuals who are multi-morbid are less likely to adhere emphasises the need for further development of interventions such as fixed dose combination pills (Selak et al., 2014). Further, barriers to adherence such as feelings of depression and anxiety could be screened for at a primary care level using validated questionnaires and addressed (DiMatte et al., 2011; Aremu et al., 2022). However, broader strategies focussing on both system and patient-specific factors will also be needed, with no individual intervention likely to be sufficient (Martin et al., 2005). Overall, it is clear that in India at least, sensitization and capacity strengthening of healthcare professionals in measuring and supporting adherence - especially in vulnerable patients including older, low literacy, suboptimal mental health and multimorbid individuals-is highly warranted.

For healthcare providers, we recommend that for these vulnerable groups especially, strategies such as the “Information-Motivation-Strategy” model for better adherence are employed (DiMatte et al., 2011). Patient-specific needs can be addressed to help achieve improved medication adherence and health outcomes through the provision of correct, patient-centred information, use of shared-decision making for better patient motivation and implementation of individualised strategies for patients to overcome barriers to adherence with the help of support from caregivers, various healthcare professionals, and peer groups (DiMatte et al., 2011; Kva et al., 2018; Aremu et al., 2022). At the health system level, we recommend policymakers utilise up to date evidence on interventions that can improve medication adherence in India and implement them to specifically target these at-risk groups—for example, through the use of community healthcare workers for those who live rurally, fixed-dose combinations for multimorbid patients and education-based interventions for those who may not fully understanding their prescribed treatments (Tolley et al., 2023).

Furthermore, the lessons from this study on design and implementation of surveys such as SAGE2 for assessing medication adherence are highly cross-applicable to future studies in India and other LMICs to inform better measurement of factors affecting medication adherence.

## Conclusion

Adherence to medications for NCDs in India is multifactorial, with patient-specific and systems-level factors interacting to influence individuals’ decision making in initiating and continuing treatment. To our knowledge, this study represents the largest nationally representative assessment of factors associated with medication adherence across NCDs in India. We recommend that future surveys examine the causes for non-adherence, specifically assess the implementation component of adherence and, attempt to distinguish behavioural and access related effects. There is a need to evaluate the evidence for interventions to improve adherence and design targeted public health measures to benefit those most at-risk of poor adherence to ensure universal health coverage.

## Data Availability

Publicly available datasets were analyzed in this study. This data can be found here: https://iipsindia.ac.in/content/SAGE-data. All code is made available on GitHub: https://github.com/KGrewal1/SAGE2Adherence.

## Abbreviations

CI: confidence interval; LMIC, low and middle income countries; NCD, non-communicable disease; OR: Odds Ratio; SAGE, Study on global AGEing and adult health; SAGE2, Second Wave of Study on global AGEing and adult health; SD, standard deviation; WHO, World Health Organisation.
