# Supplementary material for: Factors influencing adherence to non-communicable disease medication in India: secondary analysis of cross-sectional data from WHO - SAGE2
Source: Front Pharmacol. 2023 Oct 13;14:1183818. doi: 10.3389/fphar.2023.1183818 (PMC10603298; doi:10.3389/fphar.2023.1183818)
Supplement: Supplementary file 3 [file Table1.DOCX]

**Additional file 1: exact questions selected from Sage2 survey and measurement methods used**

| **Subdomain** | **Variable** | **Question** | **Details of measurement e.g. likert scale** |
| --- | --- | --- | --- |
| Socio-  demographic characteristics | Age | Q1011: “How old are you now?” | Direct question |
|  | Gender | Q1009: “Record sex of the respondent” (to be completed by the interviewer) | Direct question for interviewer: male, female and transgender options |
|  | Rural/ urban location | Q0104 | Direct question |
|  | State | Q0105a | Direct question |
|  | Religion | Q1019: “Do you belong to a religious denomination?” (The interviewer was then asked which one, if the answer was yes. The respondent replied without the categories being read.) | Direct question |
|  | Caste | Q1018: “What is your background or ethnic group?” | MCQ: scheduled tribe, scheduled caste, other backward caste, none of the above, other |
|  | Schooling | Q1015: “Have you ever been to school?” | Yes/No question |
|  | Highest level of schooling | Q1016: “What is the highest level of education that you have completed?” | MCQ |
| Economic characteristics | Wealth quintile | Based on wealth as defined in appendix 4 of the report | Income quintiles were derived from the household ownership of durable goods, dwelling characteristics. Durable  goods included number of motorbike or cars, and if, for example, the household has electricity, a television, fixed  line or mobile phone, a bucket or washing machine. A total of 21 assets were included with overlaps and  differences in the asset lists by country. |
|  | Subjective wealth | Q7002: “Do you have enough money to meet your needs?” | Likert scale |
|  | Working | Q1503: “Have you worked for at least 2 days during the last 7 days?” | Yes/No question |
| Health-related factors | Tobacco consumption | Q3001: “Have you ever smoked tobacco or used smokeless tobacco?” | Yes/No question |
|  | Alcohol consumption | Q3008: “Have you consumed alcohol in the last 30 days?” | Yes/No question |
|  | Number of Morbidities | Calculated by authors. The number of morbidities that the respondents had been diagnosed with was collated into the number of morbidities. | Direct question on all morbidities, determination of multimorbidity was carried out in this analysis |
|  | Self-reported health | Q2000: “In general, how would you rate your health today?” | Likert scale |
|  | Cognitive function | Q2010: “Overall in the last 30 days, how much difficulty did you have with concentrating or remembering things?” | None-Mild-Moderate-Severe-Extreme |
|  | Physical function | Q2002: “Overall in the last 30 days, how much difficulty did you have with moving around” | None-Mild-Moderate-Severe-Extreme |
|  | Feelings of Anxiety | Q2019: “Over the last 30 days, how much of a problem did you have with worry or anxiety?” | None-Mild-Moderate-Severe-Extreme |
|  | Feelings of Depression | Q2018: “Over the last 30 days, how much of a problem did you have with feeling sad, low or depressed?” | None-Mild-Moderate-Severe-Extreme |
|  | Cataracts | Q4062: “Since last we spoke, were you diagnosed with a cataract in one or both of your eyes (a cloudiness in the lens of the eye)?” | Yes/No question |
|  | Healthcare provider | Q5004: “Thinking about health care you needed in the last 3 years, where did you go most often when you felt sick or needed to consult someone about your health?” | MCQ |
| Community related variables | Current Marital Status | Q1012: “What is your current marital status?” | MCQ: never married, currently married, cohabiting, separated/ divorced, widowed |
|  | Community support | Q8062: “Are there children/adults in your household/compound, or living outside the household and visit regularly, that provide support or care that you need?” | Yes/No question |

Abbreviations: MCQ, Multiple Choice Question
