# Supplementary figures and images for: Factors influencing adherence to non-communicable disease medication in India: secondary analysis of cross-sectional data from WHO - SAGE2
Source: Front Pharmacol. 2023 Oct 13;14:1183818. doi: 10.3389/fphar.2023.1183818 (PMC10603298; doi:10.3389/fphar.2023.1183818)

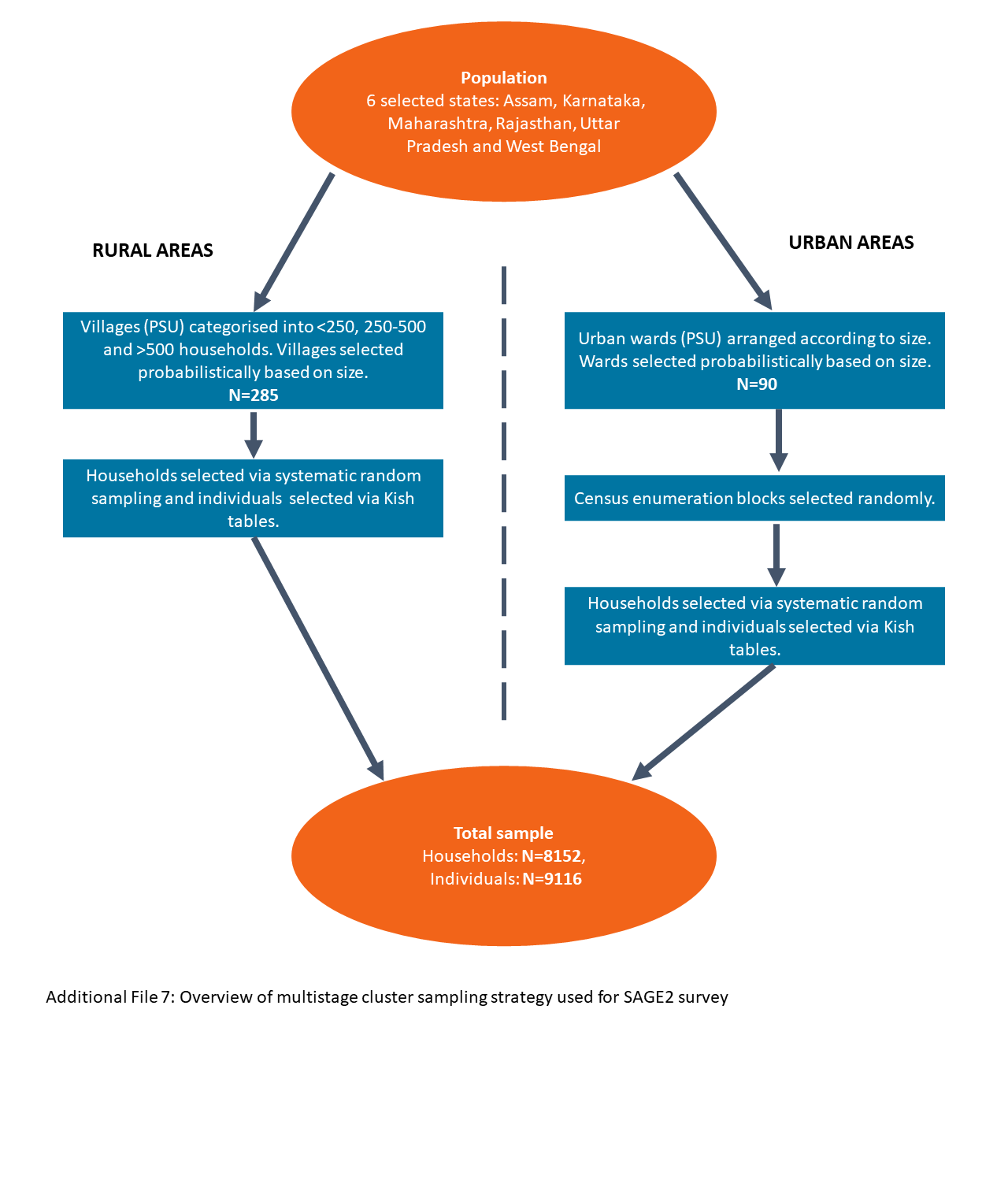

Supplement: Supplementary file 4 [file Image1.TIF]
